# Regenerative Strategy for Persistent Periprosthetic Leakage around Tracheoesophageal Puncture: Is It an Effective Long-Term Solution?

**DOI:** 10.3390/cells10071695

**Published:** 2021-07-05

**Authors:** Claudio Parrilla, Aurora Almadori, Ylenia Longobardi, Wanda Lattanzi, Marzia Salgarello, Giovanni Almadori

**Affiliations:** 1 Otolaryngology Unit, Department of Head and Neck Surgery, Fondazione Policlinico Universitario A. Gemelli IRCCS, Università Cattolica del Sacro Cuore, 00168 Rome, Italy; claudio.parrilla@policlinicogemelli.it (C.P.); ylenia.longobardi@unicatt.it (Y.L.); giovanni.almadori@policlinicogemelli.it (G.A.); 2 Plastic Surgery Unit, Department of Women’s and Child Health Sciences, Fondazione Policlinico Universitario A. Gemelli IRCCS, Università Cattolica del Sacro Cuore, 00168 Rome, Italy; marzia.salgarello@policlinicogemelli.it; 3 Centre for Nanotechnology and Regenerative Medicine, Division of Surgery and Interventional Science, University College of London, London NW3 2QG, UK; 4 Applied Biology Unit, Department of Life Sciences and Public Health, Fondazione Policlinico Universitario A. Gemelli IRCCS, Università Cattolica del Sacro Cuore, 00168 Rome, Italy; wanda.lattanzi@unicatt.it; 5 Head & Neck Oncologic Unit, Department of Head and Neck Surgery, Fondazione Policlinico Universitario A. Gemelli IRCCS, Università Cattolica del Sacro Cuore, 00168 Rome, Italy

**Keywords:** ASCs, SVF, cell therapy, fat grafting, lipotransfer, voice prosthesis, tracheoesophageal puncture, tracheoesophageal fistula, fistula closure, fistula healing

## Abstract

Autologous tissue-assisted regenerative procedures have been considered effective to close different types of fistula, including the leakage around tracheoesophageal puncture. The aim of this study was to retrospectively review 10 years of lipotransfer for persistent periprosthetic leakage in laryngectomized patients with voice prosthesis. Clinical records of patients who experienced periprosthetic leakage from December 2009 to December 2019 were reviewed. Patients receiving fat grafting were included. The leakage around the prosthesis was assessed with a methylene blue test. Twenty patients experiencing tracheoesophageal fistula enlargement were treated with fat grafting. At the one-month follow-up, all patients were considered improved with no leakage observed. At six months, a single injection was sufficient to solve 75% of cases (n 15), whereas 25% (n 5) required a second procedure. The overall success rate was 80% (n 16). Results remained stable for a follow-up of 5.54 ± 3.97 years. Fat grafting performed around the voice prosthesis, thanks to its volumetric and regenerative properties, is a valid and lasting option to solve persistent periprosthetic leakage.

## 1. Introduction

Cell therapy based on the use of mesenchymal stem cells (MSCs) found an important role in the field of tissue engineering and regenerative medicine. In particular, adipose stem cell-based regenerative strategies came under the spotlight in 2001, when Zuk et al. demonstrated for the first time that adipose tissue was abundant in multipotent stem cells, namely adipose-derived stem cells (ASCs) [1]. Compared with bone marrow, ASC yield from fat-tissue liposuction is 500-fold greater, with easier access, a less invasive harvesting procedure, fewer complications, and less damage to the donor sites [2]. Due to these practical advantages, ASC-based cell therapies have been widely applied for volumetric enhancement, wound healing, and soft tissue and bone regeneration [3,4,5,6].

In particular, autologous fat grafting (AFG) gained popularity and has been incorporated in standard clinical practice to reconstruct soft-tissue defects caused by cancer resection, trauma, and chronic wounds. AFG is mainly composed of mature adipocytes, pre-adipocytes, stem cells, and growth factors. Because of its biocompatibility, minimal invasiveness, wide availability, no immunogenicity, and great regenerative potential, AFG is now considered the ideal soft-tissue filler. Its use has been proposed in multiple clinical applications aiming at volumetric augmentation, anti-fibrotic effect, or both volumetric enhancement and tissue regeneration [7,8,9]. Among its multiple applications, AFG has been proposed to manage leakage around tracheoesophageal puncture (TEP).

After a total laryngectomy (TL), patients might easily acquire a fluent alaryngeal speech thanks to a voice prosthesis (VP), which can be implanted primarily or secondarily with respect to the TL through a TEP. Despite the disadvantages associated with VP, such as periodic replacement and careful management [10,11,12], it allows for speech restoration, making the surgical procedure more acceptable for patients and their family [13,14]. The leakage around the prosthesis represents the main cause of failure in voice prosthesis rehabilitation after TL. It may result in aspiration of liquids, foods, saliva or, sometimes, in severe displacement of the prosthesis [15]. The frequency of leakage around TEP is estimated to be 7.2% [16], and is associated with a reduced health-related quality of life. Among the possible causes, the reduction of surgical edema is an expected evolution after TEP, and granulation tissue formation, fistula enlargement, and severe tissue atrophy are less common but certainly require a more complex solution when encountered. The influence of radiotherapy in causing such complications has also been discussed [17,18]. Patients undergoing primary irradiation and/or salvage TL may experience more often clinically relevant periprosthetic leakages (PL) [19]. Furthermore, prolonged vocal effort could accelerate the reduction of the tracheoesophageal wall thickness, causing dilatation of the puncture site.

The management of PL is still a challenge and often causes multiple or prolonged hospitalization, with increased treatment costs. Management of PL includes downsizing the prosthesis, widening the esophageal flange, VP removal to enable fistula retraction, application of a purse-string suture around the fistula tract, or cauterization with silver nitrate [19]. Often these procedures have to be performed one after the other on the same patient. Although the purpose of the mentioned procedures is to narrow the fistula or to increase the tracheal and/or esophageal flanges of the prosthesis, another approach consists of augmenting the tracheoesophageal wall to increase its bulk and prevent further leakages [20]. Among various substances that can be injected to obtain a volumetric augmentation, good results can be achieved with a medical silicone elastomer implant (Vox Implant^®^, Bioplasty BV, Geleen, The Netherlands), hyaluronic acid, and autologous fat. Our team recently proposed a systematic and standardized algorithm to address this complication [15], as in the literature there is no consensus on the choice and timing of the different treatments. In our algorithm, fat grafting represents the sixth step after the aforementioned procedures.

The rationale behind using AFG to correct the leakage around TEP relies, on one hand, on increasing the tissue bulk around the prosthesis by injecting the lipoaspirate (padding effect), and on the other hand, on ameliorating the dystrophic tissues and scarring via a paracrine effect mediated by the ASCs (regenerative effect) [21]. Autologous tissue-assisted regenerative procedures, mainly based on lipotransfer, have already been explored with encouraging results to assist the closure of different types of fistula, including tracheoesophageal, pharyngo-cutaneous, and perianal fistulas [22,23,24]. So far, two case series and one case report have shown positive results after AFG to manage the leakage around TEP [25,26,27]. However, evidence on the long-term efficacy, tolerability, and cost-effectiveness of this treatment for persistent PL is currently lacking, with studies being limited by small sample size and short-term follow-up. The aim of this study is to retrospectively analyze 10 years of AFG to treat PL and to assess the presence of ASCs in the processed lipoaspirate.

## 2. Materials and Methods

### 2.1. Study Design

In this study, we selected and analyzed data related to tracheoesophageal fistula enlargement requiring AFG recorded in the last 10 years (1 December, 2009, to 31 December, 2019). The data used for this study were retrospectively gathered from existing data sources. The study was conducted according to the guidelines of the Declaration of Helsinki, and approved by the IRB (ID 3347). Written informed consent was obtained from the study participants.

The outcome was assessed as the success and complication rate of lipofilling to treat the leakage around TP. The problem was judged solved when no leakage of saliva or diluted methylene blue was reported.

### 2.2. Population

In our study, we considered all patients who accessed the otolaryngology clinic at our institution from December 2009 to December 2019 for PL. After TL, all patients are routinely trained by the speech therapist in the use and maintenance of the fistula and prosthesis, and each access to our clinic for VP-related issues is registered in a specific database. As routine practice, the speech therapist collects information in the database on the incidence, management, and outcome of adverse events experienced by patients. All the patients experiencing PL are treated with the same therapeutic approach until success is reached, with a sequence of attempts from the most to the least conservative option, taking into account patient discomfort, need for hospitalization, and costs of the procedure [9]. The sequence includes deep cleaning, prosthesis reallocation in situ, VP replacement, application of a thin silicone ring behind the tracheal flange [28], placement of a specialized VP with an enlarged flange [29], and silicone injection [30]. If the problem is still not solved, patients undergo AFG in order to thicken the TEP tract [25,26].

### 2.3. Surgical Procedure

**Figure 1:**
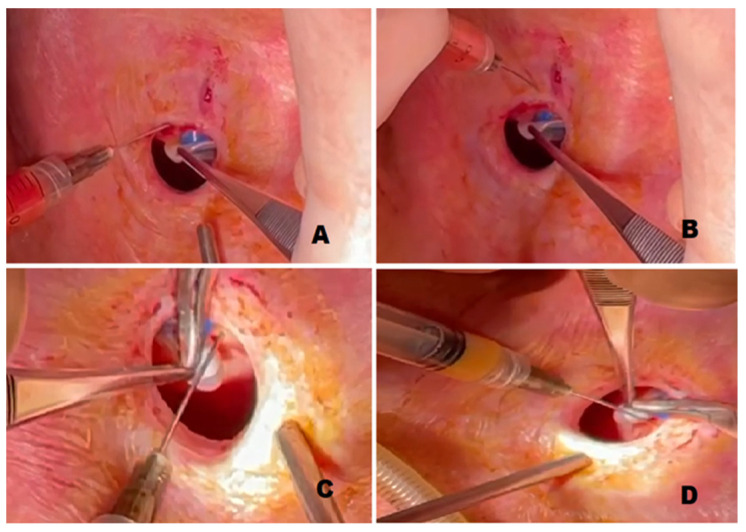
The image illustrates the sites of injection. With the voice prosthesis held in place, the lipoaspirate is injected around the prosthesis (**A**) at 9, (**B**) 12, (**C**) 3, (**D**) and 6 o’clock.

Under general anesthesia, 20 to 30 cc of adipose tissue are harvested with a Mercedes liposuction cannula (1.8 mm diameter with a bullet-like tip and 0.9 mm holes) from the superficial layer of the abdominal area [31]. The lipoaspirate is then centrifuged at 3000 rpm for 10 s to separate its components. The upper fraction, containing oil and cellular debris, and the lower fraction, containing fluids and blood, are discarded, while the middle layer, rich with ASCs and progenitor cells, is transferred into 1 mL syringes connected to a 21-gauge sharp needle. Before injection, methylene blue dye is used intra-operatively to clearly identify the leakage. Through four injection sites at 12, 3, 6, and 9 o’clock around the prosthesis, the purified lipoaspirate is injected into the tracheoesophageal wall circumferentially (Figure 1).

The voice prosthesis is held in place during the procedure. Injection is performed until the volume enhancement is considered satisfactory. Patients are usually hospitalized for 1 day after the procedure. Patients are routinely evaluated one and six months after AFG to assess the presence or absence of leakage around the prosthesis. In order to highlight any leakage, they are asked to swallow liquid methylene blue dye. Patients are then observed at a regular annual follow-up.

## 3. Results

Between December 2009 and December 2019, we recorded 661 hospital admissions from 164 patients due to PL. Out of 164 patients, 146 underwent TL with primary closure of the pharynx and 18 were reconstructed with a non-tubed free flap (14 with anterolateral thigh and 4 with forearm). All patients underwent bilateral neck dissection, myotomy of the crico-pharyngeal muscle, and TEP (primary or secondary) for voice restoration. A total of 70.73% of patients (n 116) were subjected to radiotherapy, which was primary (22 pts/13.41%) or adjuvant (94 pts/57.31%). In 60 out of 164 patients (36.58%), a secondary TEP was chosen. The interval between TL and the secondary TEP varied from 2 to 120 months (39.90 ± 12.80). All prostheses were 22.5-French VP (Provox Vega^®^, Provox XtraSeal^®^, Provox ActiValve^®^, Atos Medical AB, Horby, Sweden). Twenty out of the 164 patients (12.19%) experienced large fistula enlargement and were treated with AFG. The characteristics of patients are shown in Table 1.

**Figure 2:**
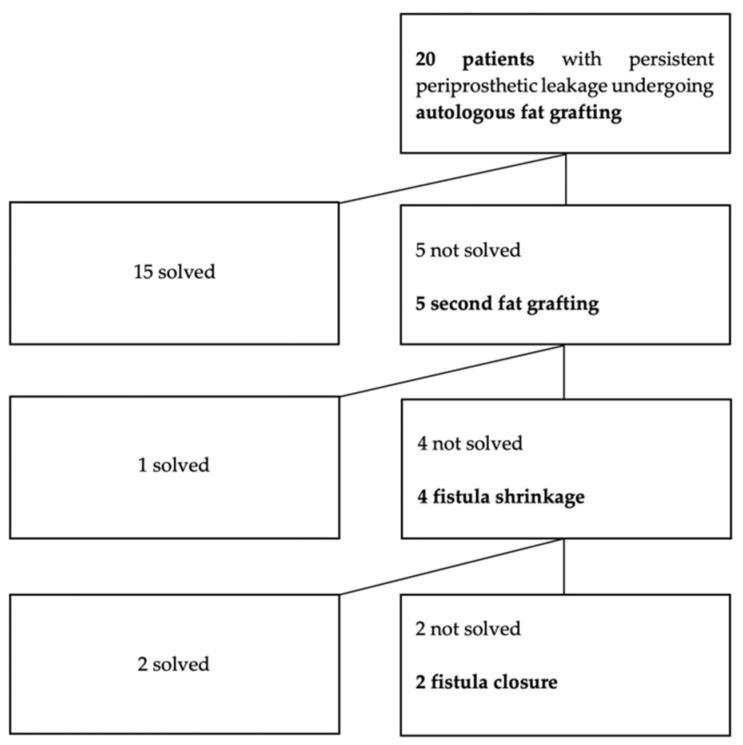
Flow chart of the 20 patients with persistent periprosthetic leakage treated with fat grafting.

**Figure 3:**
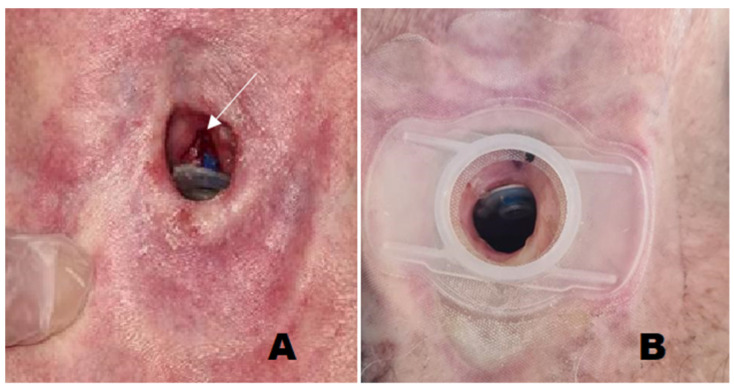
The figure illustrates an example of tracheoesophageal fistula with voice prosthesis managed with fat grafting. (**A**) The fistula is enlarged (arrow) causing leakage of saliva and food; (**B**) after fat grafting there is no leakage, as proved with a methylene blue dye test.

The 20 patients treated with AFG had experienced PL for an average time of 3.04 ± 3.58 months (range 1–6 months). Fourteen out of 20 patients (70%) showed a major fistula enlargement (8/14 = 57.14% associated with atrophic fistula; 2/14 = 14.28% associated with infected/necrotic fistula). The other six patients (30%) presented a clinically relevant leakage, defined as more than two events in a short period (1 month) associated with dilated fistula. All 20 patients had been treated with deep cleaning or reallocation of the prosthesis in situ, a VP replacement, a silicone ring placed behind the tracheal flange, placement of a specialized VP with an enlarged flange, and silicone injections, without achieving stable success over time. A total of 25 lipotransfers were performed. Patients received on average 1.25 ± 0.44 procedures. During each treatment, an average of 2.6 ± 0.5 mL was injected (SD). After treatment, short-term success (1 month after the procedure) was achieved in all patients. Longer success (>6 months) was achieved in 15 out of 20 patients (75%). Five patients experienced recurrence of PL between 1 to 5 months after the procedure. All of them underwent an additional AFG, with a mean interval time from the first procedure of 3.2 months. In one out of five, the leakage was solved, achieving long-term success (6 months after the second procedure). Overall, 16 out of 20 patients (80%) definitively solved the leakage though AFG, with a mean follow-up of 5.54 ± 3.97 years (range of 1–9 years) (Figure 2 and Figure 3).

## 4. Discussion

This study reports an effective intervention for the conservation of a voice prosthesis. Persistent PL represents the most challenging long-term complication in VP management, particularly if associated with fistula enlargement and/or fistula resorption. In these cases, simple VP downsizing or extra-flange prosthesis are not resolutive treatments and are associated with recurrences. Tracheoesophageal fistula augmentation techniques become in this subgroup of patients the only possible approach. Advantages of using synthetic products (i.e., polydimethylsiloxane elastomer implant VoxImplant^®^, Bioplasty BV, Geleen, The Netherlands, calcium hydroxylapatite Radiesse^®^, Bioform Medical, San Mateo, CA, USA, hyaluronic acid) include immediate availability and an outpatient clinic setting [20,28,29,30], but certain disadvantages limit their use. Among these, chronic inflammation and granuloma formation is associated with the use of non-resorbable biomaterials, particularly polydimethylsiloxane, and the rapid resorption rate of hyaluronic acid, broken down by hyaluronidase enzyme, requires that the procedure be repeated multiple times, with up to six injections over 18 months [20]. The advantages of using adipose tissue over other options include greater biocompatibility, no immune response, long-lasting results, and regenerative properties.

In fact, in this group of patients the main indication for AGF was to achieve volumetric restoration, but regeneration is also important to increase tissue elasticity around the VP, as patients often undergo radiotherapy for laryngeal cancer treatment. In our cohort, 80% of patients (18/20) underwent RT (Table 1). The tissue changes associated with irradiation may reduce the elasticity and integrity of TEP, and radiation-induced fibrosis (RIF) has been implicated as potential risk factors for enlarged puncture [19]. Evidence shows that fat grafting can minimize the side effects of radiation therapy by reducing RIF [32,33,34,35]. First proposed by Rigotti et al., the use of AFG has shifted the therapeutic approach of radio-damaged tissues [32]. Their results were supported by ultrastructural analysis, demonstrating improvement of fibrotic and microangiopathic radio-damaged tissues with newly formed microcirculation and reduced collagen content [32]. The authors attributed the results to the ASCs’ secretion of angiogenic factors, leading to the production of new microvessels and ameliorating circulation and tissue oxygenation [32]. In this context, the effect of AFG is not limited to leakage management by increasing the tracheoesophageal wall volumetrically, but may also have a role in preventing further failures by ameliorating the tissue quality, thereby reducing the risk of prosthesis displacement.

The results from our series are in line with previously published studies. However, the studies so far have mainly been case series or case reports (Table 2), with small sample sizes, short-term follow-ups, and lack of objective outcome assessments [25,26,27].

In our study, the volume injected was on average 2.6 ± 0.5 mL, similar to previous studies, and the number of procedures required to solve the leakage was on average 1.25, with the majority of patients requiring only one AFG. The average follow-up was 29.35 months in previous studies (Table 2) [25,26], whereas we proved a long-lasting effect for an average follow-up of 5.54 ± 3.97 years. Overall, in our series a success rate of 75% was achieved after just one injection, and with an additional injection the overall success rate rose to 80%. These data showed that although a single fat injection is effective in the majority of cases, the procedure can be repeated multiple times, allowing the success rate of the treatment to be further increased. In previous case series, the success rate was 57.14% or 60% (Table 2) [25,26]. The reason for a such difference in the outcome might be due to the fat processing technique, which mainly involves the method of harvesting and processing, aiming at potentiating the survival and functionality of ASCs [36]. Previous studies reported surgical lipectomy and manually mincing the lipoaspirate (Table 2) [25,26]. Conversely, we performed a liposuction and harvested only the superficial adipose tissue because our team had previously demonstrated that ASCs from the superficial fat layer, above the superficial fascia, present increased viability, trophism, multipotency, and stemness features [31]. We also processed the fat via centrifugation at 3000 rpm, as this is proved to be the most efficient in retaining more viable ASCs as well as to retain growth factors such as vascular endothelial growth factor (VEGF) and basic fibroblast growth factor (bFGF) [37,38], and we used a smaller needle for injection (21-gauge). In our series, we attribute the long-term success more to the regenerative properties of the ASCs rather than to the augmentative effect alone.

Although evidence of AFG on managing the leakage around TEP is limited, in the literature several reports on other applications showed that the retention rate of injected fat varies extensively, from 25–80%, likely because of differences in techniques used [39]. Optimization of the processing technique, and potentially ASC or SVF enrichment, are envisaged in the future to allow leakage resolution in more patients and with one single procedure, preventing the use of more invasive and less cost-effective procedures such as fistula shrinkage or surgical closure (Figure 2). This would be particularly relevant for the elderly and more debilitated patients. Surgical technique optimization includes the use of superficial adipose tissue, which may represent a naturally enhanced autologous filler because of the increased ASC number and biological properties [31]. The use of smaller cannulas, as proposed in the micro fat-grafting technique (14-gauge, 1 mm diameter harvesting cannula and 21-gauge, 0.8 mm diameter grafting cannula), is another approach to potentially optimize the surgical technique. It allows the selection of smaller fat lobules (500 μm in diameter) that maintain the volumetric effect but with enhanced regenerative properties [40,41,42]. The rationale for this technique is consistent with the work of Eto et al., aiming at implementing the “surviving and regenerating zones” of fat grafts composed of adipocytes and ASCs [43,44].

In addition to surgical technique optimization, cells extracted from adipose tissue can be used to enrich the lipoaspirate itself. Preclinical and clinical studies suggest that fat grafting enriched with ASCs or SVF presents an increased survival rate [45,46,47]. Laloze et al. reported retention results of 64% for enriched lipotransfer (with SVF and expanded ASCs), versus 44% for non-enriched fat grafts [48]. The advantages of enrichment with ASCs include potential differentiation into a variety of cell types such as adipocytes; abundant paracrine secretion of growth factors such as VEGF, HGF, FGF-2, and IGF-1; immunomodulation; and tolerance induction [39]. However, ASC-enriched fat grafting requires a liposuction procedure prior the primary operation, and ASC expansion also requires cultivation procedures at cell facilities, which are currently costly and time consuming. The advantages of using SVF are its easy accessibility and in-operation-room extraction, which limits the treatment to a single operation [49]. For these reasons, surgeons often prefer to apply SVF rather than ASCs. However, whereas ASCs are a homogenous cell population without cells such as leukocytes and endothelial cells, SVF cell preparations are a heterogeneous cell population composed of cell debris, perivascular cells, inflammatory cells (i.e., leukocytes), endothelial cells, and erythrocytes. Hence, the latter results in a higher immunogenicity compared to ASCs [50].

Other fat-enrichment attempts were made by adding platelet-rich plasma (PRP) to improve the vascularization and survival of the graft, because PRP contains growth factors and blood products that interact with the surrounding cells and enhance the adipogenesis, but results are contradictory [51,52,53].

Nevertheless, the relatively low number of published clinical studies and lack of standard protocols limit the application of ASC- or SVF-based cell therapy in clinical work. Whether ASC- or SVF-enriched fat grafting is effective enough to be worth the trouble is still a matter of debate, and the use of enrichment rather than conventional AFG in the leakage around the VP should be justified by superior results.

Strengths and limitations: The main strengths of the study consist of its long-term follow-up and standardized surgical methods that were not modified over the years. Despite its strengths, the study is limited by the lack of a control group to assess the efficacy, tolerability, and cost-effectiveness of this treatment over other options.

## 5. Conclusions

Thanks to the regenerative properties that alloplastic materials do not have, AFG around the voice prosthesis is a valid, long-lasting therapeutic option. The surgical technique could be optimized in order to be able to solve the leakage with a single injection; therefore, further research is required in this field. Data on the efficacy and tolerability acquired in this study can assist with the design of a future randomized clinical trial.

## Figures and Tables

**Table 1:** Demographic data.

**Characteristic**	**Patients Overall** **(n = 164)**	**Patients Undergoing Fat Grafting (n = 20)**
**Age**		
Mean ± SD	68.6 ± 7.03	70.4 ± 4.54
Range	30–81	56–72
**Sex—n (%)**		
Male	156/164 (95.12%)	20/20 (100%)
Female	8/164 (4.87%)	0/20 (0%)
**Body Mass Index (BMI)**		
Mean ± SD	24 ± 4.4	23 ± 4.1
**Pharynx closure—no (%)**		
Primary	146/164 (89.02%)	19/20 (95%)
Non-tubed free flap (anterolateral thigh)	14/164 (8.53%)	1/20 (5%)
Non-tubed free flap (forearm)	4/164 (2.43%)	0/20 (0%)
**Primary vs. Rescue Surgery—n (%)**		
Primary total laryngectomy	120/164 (73.17%)	8/20 (40%)
Rescue total laryngectomy after surgical organ preservation attempt	22/164 (13.41%)	2/20 (10%)
Rescue total laryngectomy after non-surgical organ preservation attempt	22/164 (13.41%)	10/20 (50%)
**Radiotherapy—no (%)**		
Before surgery	22/164 (13.41%)	10/20 (50%)
Adjuvant	94/164 (57.31%)	8/20 (40%)
No treatment	48/164 (29.26%)	2/20 (10%)
**Tracheoesophageal Puncture Time—no (%)**		
Primary	104/164 (63.41%)	13/20 (65%)
Secondary	60/164 (36.58%)	7/20 (35%)

**Table 2:** Review of the literature on the use of adipose stem cell-based therapies to manage tracheoesophageal puncture enlargement.

Author, Year	Processing Method	Injection	Amount Injected (mL)	N of Injection	Sample SIZE	Age (Year) Mean ± SD	RT, Dose	Follow-Up (Months) Mean ± SD	Outcome Assessment	Efficacy	Complications
Laccourreye, 2002 [25]	Harvesting with automated aspirator system (n 2); surgical fat dissection and mincing (n 5).	19-gauge needle in 4 entry points around the puncture. VP removed during injection.	Range 0.8 to 1.5	1.4 (±0.8)	7	63.3 ± 16.4 (range 41–82)	28.57% (n 2), 50 Gy 60 Gy	19.4 ± 23.7 (range 10–36)	Physician-based observation	57.14% (n 4/7)	14.29% (n 1/7) fat extrusion
Perie, 2002 [26]	Harvesting with suction machine (n 6); surgical microdissection (n 4).	Needle in 2 or 3 entry points around TEP. VP kept in place during injection.	Range 3 to 4	1.1 (±0.4)	10	64.4 (range 56–73)	90% (n 9), NR	39.3 (range 10–61)	Methylene blue liquid swallowing	60% (n 6/10)	-
Komatsubara, 2008 [27]	Harvesting with manual aspiration; processing with filtration and washing.	16-gauge needle in 3 entry points (2, 6, 10 o’clock). VP removed during injection.	1.2	2	1	68	100% (n 1) 41.4 Gy	NR	Physician-based observation	100% (n 1/1)	-

TEP—tracheoesophageal puncture, SD—standard deviation, VP—voice prosthesis, NR—not reported, Gy—gray.

## Data Availability

Data supporting the reported results are available upon request.
